# Conduction abnormalities post alcohol septal ablation for hypertrophic cardiomyopathy – A single center experience

**DOI:** 10.1016/j.ihj.2021.10.005

**Published:** 2021-10-21

**Authors:** Avinash Mani, Harikrishnan S, Sanjay G, Ajit Kumar Valaparambil

**Affiliations:** Department of Cardiology, Sreechitra Tirunal Institute for Medical Sciences and Technology (SCTIMST), Thiruvananthapuram, Kerala, India, 695011

**Keywords:** Alcohol septal ablation, Hypertrophic cardiomyopathy, Complete heart block

## Abstract

Conduction abnormalities are commonly noted after alcohol septal ablation (ASA). This was a retrospective, observational study where we studied the incidence of new onset conduction abnormalities post ASA. 23 patients, who underwent ASA over a period of 5 years, were included in the study. Baseline conduction abnormalities were noted in 26% patients (n = 6). Transient complete heart block (CHB) was noted in 21.7% (n = 5) whereas new onset right bundle branch block (RBBB) was seen in 60.8% (n = 14). Left bundle branch block was uncommon (4.3%,n = 1). Permanent pacemaker implantation was done in 4.3% (n = 1) for CHB. Conduction anomalies are frequent after ASA with RBBB being most common.

## 1 Introduction

Left ventricular outflow obstruction (LVOTO) is one of the most common manifestation of hypertrophic cardiomyopathy (HCM). Medical management with negative ionotropic drugs are considered as first-line therapy. Septal reduction therapy is recommended in drug refractory cases. Alcohol septal ablation (ASA) is a feasible percutaneous option as compared to septal myomectomy. One of the common complications after ASA is new onset conduction abnormalities. New onset right bundle branch block is commonly noted in 37–70% of patients.^1^ Rate of permanent pacemaker implantation is higher after ASA as compared to septal myomectomy and depends on procedural experience of operator.^2^^,^^3^ There is paucity of data from Eastern countries regarding procedural outcomes. The purpose of this study is to analyze the incidence and trends of new onset conduction system disturbances in patients undergoing ASA at our center.

## 2 Methods

This was a retrospective, observational, single center study which included patients undergoing ASA for HCM, over a period of 5 years. Diagnosis of HCM was based on echocardiographic features of asymmetric septal hypertrophy and LVOTO. Hospital records were analysed and echocardiographic findings, cardiac catheterization data and baseline ECG findings were recorded for all patients. Alcohol septal ablation was performed using standard technique.^4^ Post ASA, patients were monitored for new onset conduction disturbances during hospital stay and later, on routine OPD visits. Patients who developed complete heart block (CHB) had permanent pacemaker implantation (PPI) done.

All data obtained was recorded in a structured format. Categorical variables were expressed as proportions whereas continuous variables were expressed as mean ± SD. Students *t*-test was used to analyze the difference in QRS duration and PR interval pre and post ASA. Multivariable analysis was used to identify variables which could predict new onset conduction abnormalities. A p-value of <0.05 was considered to be significant.

## 3 Results

A total of 23 patients, who underwent ASA over a period of 5 years, were included in our study. The mean age of the study population was 46.1 ± 10.6 years whereas females comprised 47.6% of the study population (Table 1). Majority of patients were on beta blockers or calcium channel blockers. Baseline conduction abnormalities was noted in about a quarter of patients ranging from incomplete LBBB, RBBB to intraventricular conduction disturbance (IVCD). The average amount of alcohol used for ablation was 2.1 ± 0.8 ml 5 patients (21.7%) had transient complete heart block during the procedure, which recovered within few seconds. Significant reduction in LVOT gradient was noted after ablation in all patients (99.67 ± 33.8 vs 34.1 ± 33.5 mmHg, p < 0.05).

**Table 1:** Baseline characteristics of patients who underwent alcohol septal ablation.

Baseline characteristics	N = 23
Age in years	46.1 ± 10.6
Females, n(%)	10 (43.4)
Clinical symptoms	
Syncope	7 (30.4)
Palpitations	9 (39.1)
Baseline NYHA functional class	
NYHA II	16 (69.5)
NYHA III	7 (30.4)
History of heart failure	0 (0)
Family history of SCD	4 (17.3)
Diabetes Mellitus	1 (4.3)
Hypertension	1 (4.3)
Dyslipidemia	1 (4.3)
Medications	
Beta blocker use	22 (95.6)
CCB use	3 (13)
Baseline ECG	
PR interval, ms	163.6 ± 23.5
QRS duration, ms	101.5 ± 11.8
Conduction abnormalities:-	
Incomplete LBBB	1 (4.3)
LAHB	1 (4.3)
RBBB	1 (4.3)
IVCD	3 (13)
Baseline Echo	
Maximum LV wall thickness, mm	20.06 ± 6.02
LV ejection fraction, %	73.24 ± 8.14
LVOT gradient, mmHg	99.19 ± 35.47
MR severity	
1+	4 (17.3)
2+	13 (56.5)
3+	6 (26.1)
Cardiac Catheterization	
LVOT gradient	99.67 ± 33.8
LV end diastolic pressure	23.9 ± 9.2

CCB – calcium channel blockers, LBBB – Left bundle branch block, LAHB – Left anterior hemiblock, RBBB – right bundle branch block, IVCD – intraventricular conduction disturbance, LVOT – left ventricular outflow tract, MR – mitral regurgitation, SCD – sudden cardiac death.

Post ASA, significant increase in QRS duration was noted (130.8 ± 26.4 vs 101.5 ± 11.8, p < 0.001) whereas PR interval did not change (168.5 ± 32.9 vs 163.6 ± 23.5, P = 0.464). RBBB was the most common (60.8%) conduction abnormality post ASA (Table 2). Majority of conduction abnormalities occurred within 72 h of procedure. CHB was noted in one patient, who had IVCD at baseline and developed CHB after 48 h of procedure. He underwent PPI for the same. None of the patients had any documented ventricular tachycardia/ventricular fibrillation (VT/VF) at a mean follow up of 4.4 ± 2.2 years. Multivariable analysis did not identify any baseline variable which could predict the development of intraprocedural AV block or post procedure conduction disturbance (p > 0.05).

**Table 2:** Conduction abnormalities after ASA. RBBB – right bundle branch block, CHB – complete heart block.

Conduction changes post ablation	
QRS duration	130.8 ± 26.4
PR interval	168.5 ± 32.9
**RBBB**	14 (60.8)
<24 h	13 (56.5)
>24 h	1 (4.3)
LBBB	1 (4.3)
**CHB**	1 (4.3)
<24h	0
>24h	1 (4.3)
Pacemaker implantation	1 (4.3)

## 4 Discussion

The current study aims to evaluate the incidence of conduction abnormalities after ASA at a tertiary care center. HCM patients, with significant LVOT gradients, underwent ASA and had significant reduction in gradients. None of the patients had significant baseline conduction abnormalities.

RBBB was the most common conduction abnormality noted post ASA. This is attributed to the superior location of the fibers in the ventricular septum, which is supplied by the first septal perforator. Similar incidence of RBBB has been shown in previous studies by Valeti et al (58%)^5^ and Runquist et al (68%).^6^ New onset LBBB after ASA is uncommon, with incidence of 2–6%,^1^ similar to our study. Complete heart block is commonly seen within 24 h after ASA.^7^ In the study by El-Sabawi Bassim et al, delayed CHB, defined as CHB after 24 h, was noted in 3.2% whereas CHB after 72 h was seen in 0.8%.^7^ Delayed CHB is commonly attributed to myocardial edema post ablation, fibrosis and infarct maturation. Temporal incidence of arrhythmias needs to be evaluated as it determines the duration of hospital stay post procedure. Majority of conduction abnormalities are noted within 72 h post procedure.

The outcomes of ASA are dependent upon the technical expertise of the operators. In a study by Veselka et al, an institutional experience of >50 ASA procedures was associated with superior outcomes.^8^ Majority of data on conduction anomalies post-ASA is available from high volume centers in the West. Currently, there is a paucity of data from south-east Asian region on alcohol septal ablation outcomes. Further studies are needed to understand real world complication rates and develop a standardized protocol.

Beta blockers can be safely continued till the day before the procedure. During ablation, low volumes of alcohol (1–3 ml) is advocated, the rule of thumb being 1 ml alcohol for every 10 mm septal thickness.^9^ Development of CHB during procedure is usually an indicator for procedure termination. Majority of conduction abnormalities develop within 24 h after procedure, thus TPI can be safely removed on the day after the procedure. Intra-procedural conduction anomalies are transient and recover immediately. If new onset CHB develops and persists for >48–72 h, PPI is indicated.^10^ Currently, there is no role of drugs, including steroids, in managing new onset conduction anomalies post ASA.

The current study has its inherent limitation, it being a single center, retrospective study with a small sample size. Due to low event rates, baseline predictive factors for conduction anomalies were not identified.

## 5 Conclusion

Conduction abnormalities are common after alcohol septal ablation. RBBB is the most common conduction anomaly noted. Complete heart block and permanent pacemaker implantation rates are low after ASA. Majority of conduction anomalies develop within 72 h of ablation procedure.

## Author contribution

AM and HKS were involved in design and conceptualization of the study. AM was responsible for data collection, analysis and drafting the manuscript. HKS, SG and AKV were responsible for critical review and analysis of manuscript. The final manuscript was reviewed and approved by all the authors.

## Funding

None.

## Declaration of competing interest

The authors do not have any conflict of interests to declare.
